# Effects of Smoking on Outcomes of Antivascular Endothelial Growth Factor Therapy in Patients with Neovascular Age-Related Macular Degeneration Smoking and Anti-VEGF Therapy in nAMD

**DOI:** 10.1155/2018/2353428

**Published:** 2018-11-12

**Authors:** Hiroyuki Kamao, Katsutoshi Goto, Yumi Mito, Atsushi Miki, Junichi Kiryu

**Affiliations:** Department of Ophthalmology, Kawasaki Medical School, 577 Matsushima, Kurashiki, Okayama 701-0114, Japan

## Abstract

**Purpose:**

To evaluate the effect of smoking on the outcome of antivascular endothelial growth factor (VEGF) therapy in patients with neovascular age-related macular degeneration (nAMD).

**Methods:**

This retrospective case-control study included 64 eyes in 59 patients with treatment-naïve nAMD. Smoking habits were obtained from hospital records and patient recall. The patients were divided into ever-smokers and never-smokers. The patients were treated with ranibizumab or aflibercept for at least 1 year. Outcome measures were best-corrected visual acuity (BCVA), central retinal thickness (CRT) at the fovea, subfoveal choroidal thickness (SCT), and number of injections received.

**Results:**

There were no statistically significant differences in BCVA, CRT, or SCT changes between ever-smokers and never-smokers. The number of injections received was significantly higher in ever-smokers with a history of heavy smokers (never-smokers vs. heavy smokers: 5.3 ± 2.6/year vs. 7.3 ± 2.5/year; *P*=0.048 and mild smokers vs. heavy smokers: 5.2 ± 2.5/year vs. 7.3 ± 2.5/year; *P*=0.043). There was no significant difference in the baseline CRT or presence of atrophic retinal pigment epithelium in the fellow eyes of patients with nAMD according to smoking status; however, the baseline CRT in eyes with nAMD was significantly thinner in ever-smokers than in never-smokers (*P*=0.02).

**Conclusion:**

The anti-VEGF therapy was frequently required in nAMD patients with a history of heavy smoking. Heavy smoking could cause poor therapeutic response in nAMD patients.

## 1. Introduction

Age-related macular degeneration (AMD) [1] is a leading cause of blindness in elderly populations in developed countries, and progressive degeneration of the retinal pigment epithelium (RPE) is considered a leading factor in its pathogenesis [2]. Antivascular endothelial growth factor (VEGF) agents have improved the visual outcomes in patients with neovascular AMD (nAMD) [3]; however, patients in whom nAMD recurs or nonresponders do not gain much long-term benefit from current therapy [4]. In addition, their use is burdensome because of the frequent outpatient appointments, injections received, and the high treatment costs. Therefore, it is important to identify risk factors influencing the outcome of current therapy for nAMD patients.

Advancing age and cigarette smoking are consistently and strongly associated with the incidence of nAMD [5–11]. Cigarette smoking is a major modifiable risk factor for many chronic diseases, including a number of cardiovascular and pulmonary disorders [12, 13]. Cigarette smoke contains many chemicals that can lead to degeneration of the RPE and Bruch's membrane via accumulation of oxidative stress and reduction of choroidal blood flow [14–16]. A previous study reported that smoking affects both the incidence of nAMD and the effects of therapy; in that, there was a higher rate of recurrence of choroidal neovascularization (CNV) after laser photocoagulation in patients with nAMD who smoked than in those who did not smoke [17]. Until now, little research attention has focused on the effects of smoking on the outcome of anti-VEGF therapy in patients with nAMD. The aim of this case-control study was to determine whether cigarette smoking affects the outcome of anti-VEGF therapy in Japanese patients with treatment-naïve nAMD.

## 2. Materials and Methods

### 2.1. Study Design and Participants

We retrospectively evaluated 64 eyes in 59 Japanese patients aged 50 years or older with newly diagnosed nAMD, including typical AMD (tAMD) and polypoidal choroidal vasculopathy (PCV). The inclusion criteria were the presence of nAMD diagnosed based on the funduscopic, swept-source optical coherence tomography (OCT) (DRI OCT-1 Atlantis; Topcon Corporation, Tokyo, Japan), and angiographic findings (HRA-2; Heidelberg Engineering GmbH, Dossenheim, Germany); follow-up period of 12 months or longer after the initial intravitreal administration of ranibizumab and/or aflibercept; and a best-corrected visual acuity (BCVA) of 20/400 or better at the baseline. The retinal angiomatous proliferation was excluded due to smaller sample sizes (*n*=5). Patients who had received or were receiving other anti-VEGF agents (bevacizumab and pegaptanib) or had undergone laser photocoagulation, verteporfin photodynamic therapy, or submacular surgery were excluded, as were those with CNV as a result of high myopia, angioid streaks, hereditary disorders, or uveitis. The patients with eye diseases that could potentially influence the visual acuity of the studied eye, such as glaucoma, diabetic retinopathy, or rhegmatogenous retinal detachment, were also excluded.

### 2.2. Treatment and Assessments

All patients received intravitreal ranibizumab and/or aflibercept on a treat-and-extend basis at Kawasaki Medical School between May 2012 and March 2017. The study was approved by the Kawasaki Medical School Ethics Committee (2543-1) and was conducted according to the principles of the Declaration of Helsinki. The study is registered with the UMIN Clinical Trials Registry (UMIN000023676). All patients provided written informed consent for treatment with an anti-VEGF agent and for participation. Data on cigarette smoking were obtained from hospital records and patient recall and included pack-years, duration of smoking, and time since cessation of smoking. The patients were divided into never-smokers and ever-smokers (those who had smoked at least 1 cigarette per day for more than 1 year in their lifetime). The ever-smoker group was classified as mild smoking (<40 pack-years) and heavy smoking (≥40 pack-years); short-term smoking (<40 years) and long-term smoking (≥40 years); and short-term smoking cessation (<20 years) and long-term smoking cessation (≥20 years). Pack-years of smoking was calculated by multiplying the number of packs of cigarettes smoked per day by the number of years of smoking. One pack-year is equivalent to smoking 1 pack per day for 1 year. The primary outcome measure was the change in BCVA before (at baseline) and after (at the final visit) the start of anti-VEGF therapy. The BCVA was converted to logarithm of the minimum angle of resolution (logMAR) units for the statistical analysis. The secondary outcome measures were the retinal and choroidal thickness values at the fovea and number of injections received. The retinal and choroidal thickness values at the fovea were measured using swept-source OCT. The specifications for swept-source OCT included a 1050 nm wavelength tunable laser, an axial resolution of 8.0 *µ*m, and 100,000 A-scans per second. A three-dimensional macular scanning program with a scan density of 512 × 256 was used to measure the central retinal thickness (CRT) at the fovea on the Early Treatment Diabetic Retinopathy Study map; the mean CRT was defined as the distance between the inner limiting membrane and the outer border of the RPE in the 1 mm diameter region on the map. The CRT was automatically segmented by built-in software. Subfoveal choroidal thickness (SCT) was defined as the distance between the basal edge of the RPE and the inner surface of the sclera at the fovea. The SCT was manually measured using 96 averaged B-scan images by an experienced technician (KG) who was blinded to all patient information.

### 2.3. Data Analysis

The results are presented as the mean and standard deviation.

The Mann–Whitney *U* test was used to compare age, BCVA, retinal and choroidal thickness values, duration of follow-up, and number of injections received between the ever-smokers and never-smokers, as well as differences in the frequency of RPE atrophy in fellow eyes of patients with nAMD between the two groups. The number of injections received between ever-smokers with a history of short-term smoking cessation and ever-smokers with a history of long-term smoking cessation was compared by the Mann–Whitney *U* test. The chi-square test was used to compare proportions of sex and AMD subtype between the ever-smokers and never-smokers, as well as differences in the frequency of anti-VEGF therapy received in patients with nAMD between the two groups. The one-way ANOVA followed by Tukey's post hoc test was used to compare BCVA, retinal and choroidal thickness values, and number of injections received between never-smokers, ever-smokers with a history of mild smoking, and ever-smokers with a history of heavy smoking and between never-smokers, ever-smokers with a short-term smoking, and ever-smokers with a long-term smoking. The statistical analyses were performed using Ekuseru-Toukei 2012 (Social Survey Research Information Co., Ltd). *P* values <0.05 were considered statistically significant in all analyses.

## 3. Results

The characteristics of the patients with nAMD are shown in Table 1. The mean patient age was 73.5 ± 8.0 (range, 50–87) years. The analysis included 59 patients with nAMD in the ever-smoker group (2 women and 31 men; mean age: 72.0 ± 8.4 (range, 50–87) years) and never-smoker group (19 women and 7 men; mean age: 75.5 ± 7.0 (range, 58–86) years). The median values for pack-years of smoking, duration of smoking, and time since smoking cessation were 37.4 ± 14.8 years (22 mild smoking; 11 heavy smoking), 35.1 ± 28.2 years (15 short-term smoking; 18 long-term smoking), and 35.4 ± 30.2 years (14 short-term smoking cessation; 12 long-term smoking cessation), respectively. The two study groups were comparable for age; however, there was a significantly greater proportion of men in ever-smokers than in never-smokers (ever-smokers 93.9%, never-smokers 26.9%; *P* < 0.001).

The outcome of anti-VEGF therapy in patients with nAMD is shown in Table 2 (36 eyes in ever-smokers and 28 eyes in never-smokers). Twenty eyes had PCV and 16 eyes had tAMD in the ever-smoker group, and 11 eyes had PCV and 18 eyes had tAMD in the never-smoker group; the differences were not statistically significant (*P*=0.66). The mean BCVA was 0.28 ± 0.30 in ever-smokers and 0.37 ± 0.39 in never-smokers at baseline and 0.20 ± 0.29 and 0.29 ± 0.36, respectively, at the final visit; the difference was not statistically significant. The final BCVA was improved to a similar extent in both groups of patients. Similarly, there was no significant difference in the duration of patient follow-up and injection frequency of ranibizumab and aflibercept received between ever-smokers and never-smokers (3.6 ± 2.1 years, 5.0 ± 0.2 IVR and 14.7 ± 0.3 IVA per patient vs. 3.8 ± 2.0 years, 6.1 ± 0.2 IVR and 14.2 ± 0.4 IVA per patient). The mean SCT was 255.0 ± 118.7 *µ*m in ever-smokers and 239.2 ± 96.7 *µ*m in never-smokers at baseline and 220.8 ± 128.8 *µ*m and 201.3 ± 88.1 *µ*m, respectively, at the final visit; these differences were not statistically significant. However, the mean CRT was 298.4 ± 74.1 *µ*m in ever-smokers and 342.9 ± 77.5 *µ*m in never-smokers at baseline and 225.1 ± 60.2 *µ*m and 241.7 ± 85.1 *µ*m, respectively, at the final visit; the CRT at baseline was significantly thinner in ever-smokers than in never-smokers (*P*=0.02). The mean CRT in patients with PCV at baseline was 310.0 ± 69.4 *µ*m in ever-smokers and 316.5 ± 93.4 *µ*m in never-smokers group; these differences were not statistically significant (*P*=0.92). However, the mean CRT in patients with tAMD at baseline was 283.7 ± 79.4 *µ*m in ever-smokers and 358.6 ± 62.7 *µ*m in never-smokers (*P*=0.004); the CRT at baseline was significantly thinner in patients with tAMD who smoked. Atrophy of the outer retina and/or reduction of macular edema were listed as causes of the CRT thinning in the ever-smokers. To investigate this relationship further, we compared the CRT and differences in the frequency of RPE atrophy in the fellow eyes of the patients with nAMD between the ever-smokers and never-smokers. The mean CRT in the fellow eyes in patients with nAMD, PCV, and tAMD was 232.2 ± 20.7 *µ*m, 232.3 ± 21.0 *µ*m, and 232.0 ± 21.4 *µ*m in ever-smokers and 231.1 ± 18.0 *µ*m, 228.2 ± 24.4 *µ*m, and 232.9 ± 14.2 *µ*m in never-smokers, respectively, with no significant between group differences. The frequency of RPE atrophy in the fellow eyes of patients with nAMD, PCV, and tAMD was 21.7%, 16.7%, and 27.3% in ever-smokers and 35.7%, 20.0%, and 44.4% in never-smokers, respectively, with no significant between group differences.

To investigate the effect of different smoking habits, smoking intensity, smoking duration, and duration of smoking cessation, on the outcome of anti-VEGF therapy, we compared BCVA, CRT, SCT, and number of injections received between never-smokers, ever-smokers with a history of mild smoking, and ever-smokers with a history of heavy smoking; never-smokers, ever-smokers with a history of short-term smoking, and ever-smokers with a history of long-term smoking; and ever-smokers with a history of short-term smoking cessation and ever-smokers with a history of long-term smoking cessation (Table 3). There were no significant differences in duration of smoking and time since cessation of smoking. However, the mean number of injections received in never-smokers, ever-smokers with a history of mild smoking, and ever-smokers with a history of heavy smoking was 5.3 ± 2.6 per year, 5.2 ± 2.5 per year, and 7.3 ± 2.5 per year, respectively; the number of injections received was significantly higher in ever-smokers with a history of heavy smokers (never-smokers vs. heavy smokers: 5.3 ± 2.6/year vs. 7.3 ± 2.5/year; *P*=0.048, mild smokers vs. heavy smokers: 5.2 ± 2.5/year vs. 7.3 ± 2.5/year; *P*=0.043). There was no significant difference in the injection frequency of ranibizumab and aflibercept received between never-smokers, ever-smokers with a history of mild smoking, and ever-smokers with a history of heavy smoking (6.1 ± 0.2 IVR and 14.2 ± 0.4 IVA per patient, 3.8 ± 0.3 IVR and 12.0 ± 0.4 IVA per patient, and 7.6 ± 0.6 IVR and 19.9 ± 0.9 IVA per patient, respectively)

## 4. Discussion

Of the many modifiable risk factors for nAMD, cigarette smoking is the one consistently associated with the incidence of the disease. Three large population-based cohort studies [6–8], conducted mainly in European populations, reported that cigarette smoking increased the risk of incidence of nAMD by 2- to 4-fold. A report from the Surgeon General of the US Public Health Service states that there is now sufficient evidence of a causal relationship between smoking and incidence of nAMD [18]. A strong association was found between cigarette smoking and incidence of nAMD in Asia, which was independent of racial variations in the prevalence or frequencies of subtypes of nAMD [19, 20]. Moreover, several epidemiologic studies performed in Asia documented higher prevalence of late AMD in men than in women, which is attributed to the substantially higher prevalence of smoking in men than in women in Asian countries [21–23]. In the present study, there was a significantly greater proportion of men in the ever-smokers, and our results are consistent with previous epidemiologic studies suggesting the harmful effects of smoking in the eyes. Previous studies have similarly reported that smoking not only increases the risk of incidence of nAMD but also affects the outcome of therapeutic interventions. Maguire et al. reported that a higher recurrence rate of choroidal neovascular membrane after laser photocoagulation in patients with nAMD were classified as heavy-smokers (≥10 cigarettes/day) than in their mild smoker counterparts (<10 cigarettes/day). Although anti-VEGF agents can improve the visual outcome in patients with nAMD and are now used first-line in this disease, patients who develop recurrent disease and those who do not respond initially are unlikely to derive much long-term benefit from anti-VEGF therapy, and many patients eventually become dissatisfied with the outcomes of this treatment. Thus, it is important to investigate the relationship between smoking and the efficacy of anti-VEGF therapy in patients with the disease. To our knowledge, ours is the first case-control study to investigate this relationship in Japanese patients with nAMD. We investigated the visual acuity, retinal thickness, choroidal thickness, and number of injections received in patients with treatment-naïve nAMD classified according to smoking status before and after anti-VEGF therapy. We found an evidence of a difference in changes in number of injections received between heavy smokers and mild/nonsmokers. Our results are consistent with the previous report of the harmful therapeutic effects of smoking in patients with nAMD. The general population is unaware of the strong risk factor for the incidence of nAMD and the therapeutic effects for the patients with the disease; therefore, these hazards of smoking should be highlighted to the public. Previous studies demonstrated the effect of smoking cessation on the incidence of AMD [24, 25]. Delcourt et al. reported that the risk of late AMD including nAMD and geographic AMD increases until 20 years after smoking cessation. Tan et al. reported that smoking cessation reduces the incidence of late AMD. In this study, there was no significant therapeutic difference between short-term smoking cessation group and long-term smoking cessation group, and the current smoker group is of smaller sample size (8 eyes in 7 patients). Although our results do not permit us to show evidence on the beneficial therapeutic effect of smoking cessation, we are able to advise not to smoke due to prevention of the incidence of nAMD in the fellow eye.

In this study, we found that the baseline CRT in eyes with nAMD was significantly thinner in ever-smokers than in never-smokers and the thinning was not observed in ever-smokers with PCV but in ever-smokers with tAMD. Cigarette smoke contains more than 4000 chemicals that can be divided into two phases, i.e., a particulate phase that includes nicotine, tar, and benzopyrene and a gaseous phase that includes carbon monoxide, oxides of nitrogen, and hydrogen cyanide [26]. These compounds cause oxidative damage and ischemia in response to arteriosclerosis in almost all tissues in the body and induce many chronic diseases, including cerebral stroke, myocardial infarction, and chronic obstructive pulmonary disease. The mechanism of CNV is not fully elucidated; however, one pathway via which smoking promotes the development of CNV is by impairment of the RPE and Bruch's membrane because of accumulated oxidative stress and decreased blood flow in the choriocapillaris. Moreover, recent investigations of the acute effects of smoking have demonstrated a reduction in retinal blood flow as a result of vasoconstriction [27], an increase in leukocyte velocity in the macula [28] and optic nerve head [29], and a transient reduction in choroidal thickness [30], whereas the chronic effects of smoking did not include changes in retinal or choroidal thickness [31] in healthy subjects. However, there are few reports on the chronic effects of smoking in patients with nAMD. In the present study, the mean CRT was defined as the distance between the inner limiting membrane and the outer border of the RPE in a 1 mm diameter region centered on the fovea, so the CRT included both the outer retina and macular edema. We hypothesized that the CRT thinning observed in patients with nAMD who were ever-smokers was caused by thinning of the outer retina and/or reduction of macular edema and that the thinning of the outer retina resulted from the outer retinal atrophy directly or secondary to RPE atrophy. The outer retinal thickness and presence of RPE atrophy in eyes with nAMD could not be evaluated because of bleeding and macular edema, so the outer retinal thickness was assessed by measurement of CRT in the fellow eyes of patients with nAMD and the frequency of RPE atrophy was assessed by the presence of RPE atrophy in the fellow eyes of these patients using fundus autofluorescence. No difference in outer retinal thickness or frequency of RPE atrophy was found between ever-smokers and never-smokers. In addition, no difference in choroidal thickness was detected between ever-smokers and never-smokers in the nAMD group, indicating that smoking does not contribute to atrophy of the RPE. Therefore, the main cause of the CRT thinning in patients with nAMD who smoke could be reduction of macular edema, which, in turn, could be attributed to reduction of CNV leakage. Previous histopathological studies in nAMD patients demonstrated that there is a histological difference in CNV between PCV and tAMD. The CNV of tAMD was smooth muscle cell- and fibroblast-rich granulation tissue [32–34], whereas the choroidal vascular abnormalities of PCV were dilated vessels with extravasation of plasma protein and hyaline tissue [35]. The nicotine contained in cigarette induces vasoconstriction by contraction in vascular smooth muscle; therefore, smoking causes reduction of CNV leakage by vasoconstriction and/or decreased blood flow in the choriocapillaris due to arteriosclerosis. Many research studies on the effects of smoking in patients with nAMD were done to verify these hypotheses.

This study has some potential limitations. One limitation is the degree of selection bias that may have been introduced by inclusion of smokers, who are more likely to drop out of studies because of serious illness (e.g., pulmonary, coronary, or cerebrovascular disease) or death. In our study, the proportions of ex-smokers and current smokers were 80.8% and 19.2% in the nAMD group and 50.0% and 50.0% in a healthy control subjects (data not shown), respectively. This distribution is likely to reflect the fact that patients with nAMD who are current smokers are at higher risk of serious illness or death and are more likely to cease anti-VEGF therapy or outpatient appointments. However, whether this is genuinely a source of bias could only be confirmed by longer-term follow-up. Another limitation is the CNV lesions bias that may have affected CRT. PCV is characterized by wide-spread branching vascular network with multiple polypoidal lesions; therefore, the location and number of polyps may affect CRT. In this study, the CRT in eyes with no PCV but tAMD was significantly thinner in ever-smokers than in never-smokers. Since these patients were first-onset and treatment-naïve tAMD patients and we have performed laser photocoagulation for tAMD with extrafoveal CNV, the location of CNV lesions has limited effect on CRT.

## 5. Conclusions

Heavy smoking increased number of injections received in patients with treatment-naïve nAMD. Patients with nAMD who smoked had a thinner baseline CRT than those who did not, which was attributed to reduction of macular edema. Our present findings contribute additional information on anti-VEGF therapy in patients with nAMD and should heighten awareness about the risks of smoking amongst both the general population and health professionals.

## Figures and Tables

**Table 1:** Characteristics of patients with neovascular age-related macular degeneration patients.

	Ever-smokers (*n*=33)	Never-smokers (*n*=26)	*P*
Age (years), mean (SD)	72.0 (8.4)	75.5 (7.0)	0.10^*∗*^
Sex, no. (%)			
Men	31 (94)	7 (27)	
Women	2 (6)	19 (73)	<0.001^‡^
Pack-years of smoking, no. (%)			
<40	22 (67)	—	
40≤	11 (33)	—	
Duration of smoking (yrs), no. (%)			
<40	15 (45)	—	
40<	18 (55)	—	
Time since cessation of smoking (yrs), no. (%)			
<20	14 (54)	—	
20≤	12 (46)	—	

^*∗*^Mann–Whitney *U* test; ^‡^chi-square test.

**Table 2:** Results of anti-VEGF therapy in eyes with neovascular age-related macular degeneration.

	Ever-smokers (*n*=36)	Never-smokers (*n*=28)	*P*
AMD subtype, no. (%)			
PCV	20 (56)	11 (39)	
tAMD	16 (44)	17 (61)	0.66^‡^
Baseline, mean (SD)			
VA (logMAR)	0.28 (0.30)	0.37 (0.39)	0.48
Central retinal thickness (*µ*m)	298.4 (74.1)	342.1 (77.5)	0.02
PCV	310.1 (69.4)	316.5 (93.4)	0.92
tAMD	283.7 (79.4)	358.6 (62.7)	0.004
Subfoveal choroidal thickness (*µ*m)	255.0 (118.7)	239.2 (96.7)	0.8
Outcome, mean (SD)			
VA (logMAR)	0.20 (0.29)	0.29 (0.36)	0.23
Central retinal thickness (*µ*m)	225.1 (60.2)	241.7 (85.1)	0.56
Subfoveal choroidal thickness (*µ*m)	220.8 (128.8)	201.3 (88.1)	0.90
Change, mean (SD)			
VA (logMAR)	−0.07 (0.32)	−0.08 (0.39)	0.82
Central retinal thickness (*µ*m)	73.2 (85.4)	100.4 (100.2)	0.14
Subfoveal choroidal thickness (*µ*m)	31.2 (46.6)	38.0 (38.8)	0.67
Follow-up (yrs)	3.6 (2.1)	3.8 (2.0)	0.60
No. of injections (no./yr)	5.9 (2.7)	5.3 (2.6)	0.30
IVR (no./patient)	5.0 (0.2)	6.1 (0.2)	
IVA (no./patient)	14.7 (0.3)	14.2 (0.4)	0.75^‡^

^‡^Chi-square test.

**Table 3:** Results of Anti-VEGF therapy in eyes with neovascular age-related macular degeneration.

	0 (*n*=28)	Pack-years of smoking		Duration of smoking (yrs)		Time since cessation of smoking (yrs)	
		<40 (*n*=24)	40≤ (*n*=12)	<40 (*n*=16)	40≤ (*n*=20)	<20 (*n*=16)	20≤ (*n*=12)
Outcome, mean (SD)							
VA (logMAR)	0.29 (0.36)	0.24 (0.32)	0.14 (0.23)	0.14 (0.23)	0.25 (0.34)	0.24 (0.31)	0.12 (0.26)
Central retinal thickness (*µ*m)	241.7 (85.1)	216.8 (50.7)	241.8 (75.7)	223.8 (52.5)	226.3 (67.1)	240.6 (75.2)	219.8 (42.0)
Subfoveal choroidal thickness (*µ*m)	201.3 (88.1)	241.1 (135.4)	189.3 (111.8)	269.4 (150.3)	187.4 (97.8)	173.9 (96.0)	249.8 (147.1)
Change, mean (SD)							
VA (logMAR)	−0.08 (0.39)	−0.03 (0.33)	−0.15 (0.28)	−0.07 (0.17)	−0.08 (0.40)	−0.08 (0.32)	−0.06 (0.19)
Central retinal thickness (*µ*m)	100.4 (100.2)	87.4 (72.0)	44.8 (105.1)	63.4 (58.2)	81.1 (103.0)	66.31 (102.2)	62.3 (66.1)
Subfoveal choroidal thickness (*µ*m)	38.0 (38.8)	29.1 (39.4)	15.3 (56.9)	39.9 (38.9)	24.3 (51.9)	30.31 (46.8)	40.8 (38.6)
No. of injections (no./yrs)	5.3 (2.6)	5.2 (2.5)	7.3 (2.5)^*∗∗*^	5.2 (2.7)	6.5 (2.6)	6.9 (2.2)	5.5 (2.7)
IVR (no./patients)	6.1 (0.2)	3.8 (0.3)	7.6 (0.6)	2.9 (0.3)	6.8 (0.4)	6.8 (0.5)	2.7 (0.4)
IVA (no./patients)	14.2 (0.4)	12.0 (0.4)	19.9 (0.9)	12.4 (0.6)	16.5 (0.5)	17.6 (0.6)	14.6 (0.9)

^*∗∗*^One-way ANOVA followed by Tukey's post hoc test.

## Data Availability

The data used to support the findings of this study are restricted by the Kawasaki Medical School Ethics Committee in order to protect patient privacy. Data are available from Hiroyuki, Kamao, MD, PhD (hironeri@med.kawasaki-m.ac.jp) for researchers who meet the criteria for access to confidential data.
